# Autologous blood transfusion in acute type A aortic dissection decreased blood product consumption and improved postoperative outcomes

**DOI:** 10.1016/j.xjon.2022.07.005

**Published:** 2022-07-20

**Authors:** Elizabeth L. Norton, Karen M. Kim, Shinichi Fukuhara, Katelyn P. Monaghan, Aroma Naeem, Xiaoting Wu, Gorav Ailawadi, Himanshu J. Patel, G. Michael Deeb, Bo Yang

**Affiliations:** a Division of Cardiothoracic Surgery, Department of Surgery, Emory University School of Medicine, Atlanta, Ga; b Department of Cardiac Surgery, Michigan Medicine, Ann Arbor, Mich

**Keywords:** acute type A aortic dissection, aorta, blood transfusion, autologous blood transfusion, ABT, autologous blood transfusion, ATAAD, acute type A aortic dissection, CPB, cardiopulmonary bypass, FFP, fresh frozen plasma, INR, international normalized ratio, MPS, malperfusion syndrome, PRBCs, packed red blood cells, STS, Society of Thoracic Surgeons

## Abstract

**Objective:**

To evaluate the effect of autologous blood use on blood product consumption and outcomes after acute type A aortic dissection repair.

**Methods:**

From 2010 to October 2020, 497 patients underwent open acute type A aortic dissection repair, including those with autologous blood harvesting before cardiopulmonary bypass and transfusion after cardiopulmonary bypass (autologous blood transfusion [ABT], n = 397) and without autologous blood harvesting and transfusion (No-ABT, n = 100). The median ABT volume was 900 mL. Using propensity score matching, 89 matched pairs were identified based on age, sex, body mass index, preoperative hemoglobin, acute preoperative stroke, previous cardiac surgery, and cardiogenic shock.

**Results:**

After propensity score matching, both groups were similar in demographic characteristics and aortic procedures. The ABT group required significantly less intraoperative transfusion of blood products (6 vs 11 units; *P* < .0001), including packed red blood cells (2 vs 4), fresh frozen plasma (2 vs 4), platelets (2 vs 2), and cryoprecipitate (0 vs 1); and combined intraoperative and postoperative transfusion (9 vs 13; *P* < .001). ABT was protective against intra- and postoperative blood product transfusion (odds ratio, 0.28; *P* = .01). The ABT group had significantly less sepsis, acute renal failure requiring dialysis, reintubation, and shorter intubation times and postoperative lengths of stay. Operative mortality was 6.7% in the ABT group versus 13% in the No-ABT group (*P* = .14). The midterm survival was similar between the 2 groups (5 year: 76% vs 74%). ABT had a hazard ratio of 0.81 for midterm mortality (*P* = .41).

**Conclusions:**

Autologous blood transfusion was associated with better short-term outcomes and could be used routinely for acute type A aortic dissection repair. External multicenter prospective validation would be warranted.

## Graphical abstract

Autologous blood harvesting and transfusion was safe and improved short-term outcomes in patients undergoing open ATAAD repair. All patients undergoing open ATAAD repair could have autologous blood transfusion unless they are unstable needing immediate CPB or severely anemic (hemoglobin <9 g/dL).

**Figure:**
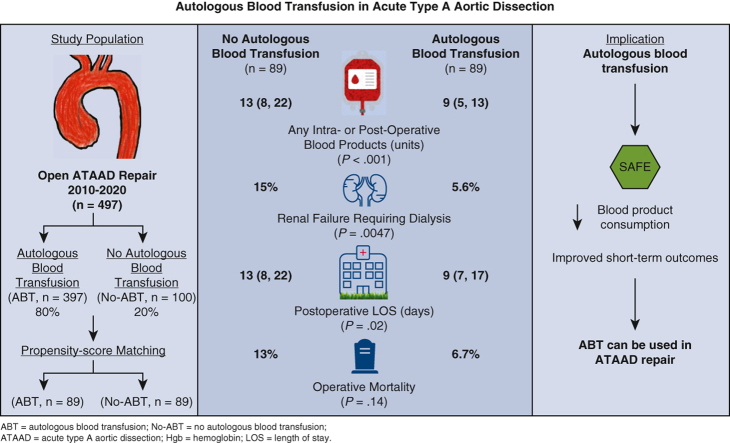

**Figure:**
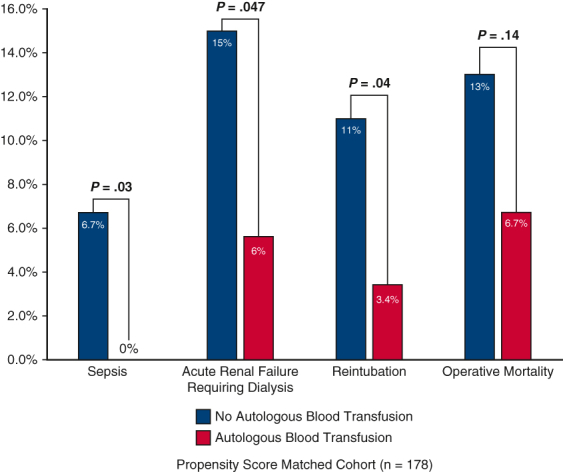
Autologous blood transfusion improved short-term outcomes.

**Central Message**

Autologous blood harvesting and transfusion was safe and improved short-term outcomes in ATAAD patients undergoing open aortic repair.

**Perspective**

Autologous blood harvest and transfusion was safe and improved short-term outcomes in ATAAD patients after open repair. All ATAAD patients could have autologous blood transfusion unless with they were hemodynamically unstable or severely anemic preventing autologous blood harvesting.

Cardiac surgery is associated with perioperative blood loss and disturbances in the coagulation system from cardiopulmonary bypass (CPB) and hypothermia resulting in perioperative anemia. The preferred treatment of this perioperative anemia has been transfusion. However, transfusion has been associated with increased morbidity and mortality.^1^, ^2^, ^3^ In addition, transfusion has risks such as acute hemolytic transfusion reactions, transfusion-related acute lung injury, transfusion-related circulatory overload, and transfusion-transmitted viral infections. Cardiac surgery contributes to overall consumption of the limited blood products.^4^ In acute type A aortic dissection (ATAAD) repair, 93.7% of patients receive some form of blood product in a recent Society of Thoracic Surgeons (STS) Database study.^5^ The average requirement is 5 U packed red blood cells (PRBCs), 4 U fresh frozen plasma (FFP), 1 U cryoprecipitate, and 3 U platelets.^5^ As a result, blood conservation strategies are becoming increasingly employed, including autologous blood transfusion (ABT). ABT has been associated with decreased blood product consumption in patients undergoing cardiac surgery.^6^, ^7^, ^8^ However, it is unknown if it is possible to harvest autologous blood from patients with an ATAAD before CPB because there is always a concern of aortic rupture causing bleeding and debate whether or not ABT is beneficial to patients with ATAAD undergoing emergency open repair.

In the past 10 years, we have been practicing harvesting autologous blood from patients with ATAAD as we do for patients undergoing elective cardiac surgery. We hypothesized that ABT in patients with ATAAD could improve the short- and midterm outcomes in ATAAD repair.

## Methods

This study was approved by the Institutional Review Board at the University of Michigan, Michigan Medicine (HUM00119716, September 27, 2016), a waiver of consent was obtained, and was in compliance with Health Insurance Portability and Accountability Act regulations.

### Study Population

Between January 2010 and October 2020, 497 patients underwent open repair of an ATAAD, of whom 80% (n = 397) received ABT and 20% (n = 100) did not receive autologous blood transfusion (No-ABT).

Investigators leveraged the STS data elements from the University of Michigan Cardiac Surgery Data Warehouse to identify the cohort and determine preoperative, operative, and postoperative characteristics. Electronic medical records were reviewed to supplement data collection. Investigators utilized the National Death Index database through June 30, 2020,^9^ medical record review, and telephone call survey (including letters and telephone calls) to obtain midterm survival. Midterm follow-up for survival status was 100% complete during our study period.

### Autologous Blood Harvesting and Transfusion

In hemodynamically stable patients, after anesthetic induction and intubation, autologous whole blood was withdrawn from the central venous line that was placed in the internal jugular vein or femoral vein by our anesthesia team before the commencement of CPB. EDTA was used for anticoagulation for autologous blood. In patients with cardiac tamponade and hypotension, the cardiac tamponade was released partially or completely first. Once patients became stable, the autologous blood was harvested. In patients requiring inotropes the autologous blood was still harvested, as long as patients could tolerate the harvesting. In patients hemodynamically stable with malperfusion and subsequent malperfusion syndrome (MPS), the malperfusion syndrome was first resolved endovascularly^10^ followed by open aortic repair following recovery from MPS and organ failure. In those patients with resolved MPS, autologous blood was harvested routinely. Patients with malperfusion but no MPS underwent emergency open aortic repair and autologous blood was harvested routinely. The absolute contraindications for autologous blood were aortic rupture or cardiac arrest needing immediate CPB, preoperative hemoglobin <9 g/dL, or unstable hemodynamic parameters. Otherwise, it was surgeons' and anesthesiologists' preference. We usually harvest 3 U autologous blood for most patients, more for Jehovah's Witness patients if tolerated. Following termination of CPB, after patients were decannulated and protamine was given, autologous blood was transfused back to the patient.

The operative strategy in ATAAD patients has been previously described.^11^, ^12^, ^13^

### Statistical Analysis

Descriptive statistics were performed to provide information on the demographic, clinical, and surgical characteristics. Continuous variables were summarized by median (25th, 75th percentile) and categorical variables were reported as n (%) in the descriptive tables. Univariate comparisons between ABT and No-ABT groups were performed using χ^2^ tests or Fisher exact tests for categorical data and Wilcoxon rank sum tests for continuous data. Among all patients, multivariable logistic regression was used to assess the effect of autologous blood use for any (intra- or postoperative) blood transfusion adjusting for age, sex, body mass index, preoperative renal failure, peripheral vascular disease, cardiogenic shock, previous cardiac surgery, connective tissue disease, and preoperative hemoglobin level. Among all patients, multivariable logistic regression was also used to assess the effect of autologous blood use for operative mortality, adjusting for age, sex, connective tissue disease, preoperative renal failure, chronic obstructive pulmonary disease, preoperative acute paralysis, preoperative acute stroke, cardiogenic shock, and previous cardiac surgery. Crude survival curves since operation were estimated using the nonparametric Kaplan-Meier method. Log-rank test was used to compare the survival between groups. Cox proportional hazard regression was performed to calculate the hazard ratio (HR) for midterm mortality for all patients from operation by adjusting group, sex, chronic obstructive pulmonary disease, previous cardiac surgery, preoperative renal failure, preoperative acute paralysis, preoperative acute stroke, connective tissue disease, diabetes, and cardiogenic shock. The covariates for the logistic and Cox models were selected based on clinical judgment and the difference between the autologous blood groups in the univariate analysis. The models had *C* statistics between 0.72 and 0.81, which indicated good model performance and that overfitting was not an issue.

Propensity score matching was performed with 1:1 matching based on age, sex, body mass index, preoperative hemoglobin level, preoperative acute stroke, previous cardiac surgery, and cardiogenic shock. Caliper matching was performed using a 0.1 SE of the logit propensity score. The matching yielded 89 matching pairs. Balance of each variable between the 2 matching groups was validated with standardized mean difference. Standardized mean difference ≤0.1 were considered a negligible difference between the 2 matching groups. All statistical calculations used SAS version 9.4 (SAS Institute) and were considered significant at *P* < .05.

## Results

### Demographics/Preoperative Data

Overall, the ABT group was younger (59 vs 62 years; *P* = .02), more likely men (70% vs 57%; *P* = .02), had higher body mass index (28.7 vs 27.4; *P* = .04), less connective tissue disease (2.3% vs 7.0%; *P* = .03) and cardiogenic shock (7.3% vs 24%; *P* < .0001), and lower preoperative hemoglobin (11.6 vs 12.6 g/dL; *P* < .0001) and hematocrit (35% vs 38%; *P* = .0003) levels compared with the No-ABT group. Other preoperative characteristics, including hypertension, history of renal failure, previous cardiac surgery, acute renal failure, malperfusion syndrome, preoperative platelet level, international normalized ratio (INR), and preoperative anticoagulant and aspirin use were similar between groups.

Following propensity score matching, both groups were similar except for the ABT group having more diabetes (11% vs 1.1%; *P* = .005) and slightly lower preoperative INR (1.0 vs 1.1; *P* = .04). (Table 1). The median platelet counts and INR were in the normal range in both groups (Table 1). There was 1 patient taking a platelet inhibitor in the ABT group and 0 patients in the No-ABT group.

**Table 1:** Demographic characteristics and preoperative comorbidities

Variable	Total (N = 497)	No-ABT (n = 100)	ABT (n = 397)	*P* value∗	PSM No-ABT (n = 89)	PSM-ABT (n = 89)	*P* value∗
Characteristic							
Patient age (y)	60 (51, 68)	62 (53, 72)	59 (50, 67)	**.02**	61 (52, 71)	61 (50, 69)	.52
Male sex	333 (67)	57 (57)	276 (70)	**.02**	52 (58)	55 (62)	.65
BMI	28.2 (25.1, 32.8)	27.4 (24.5, 30.7)	28.7 (25.2, 33.2)	**.04**	27.7 (24.6, 30.8)	27.7 (24.6, 31.4)	.49
Preexisting comorbidities							
Hypertension	384 (77)	74 (74)	310 (78)	.38	65 (73)	69 (78)	.49
Diabetes	42 (9.9)	4 (4.0)	38 (9.6)	.07	1 (1.1)	10 (11)	**.005**
Smoking status				.21			.51
Never	197 (40)	47 (47)	150 (38)	.08	43 (48)	35 (40)	.25
Former	127 (26)	23 (23)	104 (26)	.54	19 (21)	21 (24)	.69
Current	171 (35)	29 (29)	142 (36)	.22	27 (30)	32 (36)	.40
CAD	80 (16)	18 (19)	62 (16)	.51	14 (16)	13 (15)	.81
COPD	59 (12)	15 (15)	44 (11)	.28	13 (15)	8 (9.0)	.25
History of MI	29 (5.8)	8 (8.0)	21 (5.3)	.30	8 (9.0)	2 (2.2)	.051
History of renal failure	20 (4.0)	7 (7.0)	13 (3.3)	.15	7 (7.9)	4 (4.5)	.35
History of CVA	23 (4.6)	7 (7.0)	16 (4.0)	.28	7 (7.9)	4 (4.5)	.35
PVD	146 (29)	26 (26)	120 (30)	.41	22 (25)	27 (30)	.40
CTD	16 (3.2)	7 (7.0)	9 (2.3)	**.03**	6 (6.7)	3 (3.4)	.30
Previous cardiac surgery	37 (7.4)	12 (12)	25 (6.3)	.052	12 (13)	9 (10)	.49
Preoperative AI				.99			.86
None	118 (25)	25 (26)	93 (24)	.71	23 (27)	18 (20)	.31
Trace	53 (11)	10 (10)	43 (11)	.83	8 (9.4)	10 (11)	.67
Mild	133 (28)	27 (28)	106 (28)	.92	23 (27)	28 (32)	.49
Moderate	81 (17)	16 (17)	65 (17)	.95	14 (16)	14 (16)	.92
Severe	95 (20)	18 (19)	77 (20)	.77	17 (20)	18 (20)	.94
Ejection fraction (%)		58 (55, 65)	58 (55, 65)	.62	59 (55, 65)	60 (55, 65)	.68
Acute MI	20 (4.0)	6 (6.0)	14 (3.5)	.26	3 (3.4)	2 (2.2)	.65
Acute stroke	44 (8.9)	10 (10)	34 (8.6)	.65	10 (11)	9 (10)	.81
Acute renal failure	40 (8.0)	11 (11)	29 (7.3)	.22	9 (10)	9 (10)	1.0
Acute paralysis	12 (2.4)	2 (2.0)	10 (2.5)	.76	1 (1.1)	4 (4.5)	.37
Cardiogenic shock	53 (11)	24 (24)	29 (7.3)	**<.0001**	15 (17)	19 (21)	.45
Preoperative creatinine	1.0 (0.8, 1.3)	1.1 (0.8, 1.4)	1.0 (0.8, 1.3)	.41	1.1 (0.8, 1.4)	1.0 (0.8, 1.3)	.86
Malperfusion syndrome	124 (25)	32 (32)	92 (23)	.07	27 (30)	26 (29)	.87
Coronary	21 (4.2)	6 (6.0)	15 (3.8)	.40	3 (3.4)	3 (3.4)	1.0
Cerebral	42 (8.5)	9 (9.0)	33 (8.3)	.83	9 (10)	9 (10)	1.0
Spinal cord	8 (1.6)	3 (3.0)	5 (1.3)	.20	2 (2.2)	2 (2.2)	1.0
Celiac	9 (1.8)	1 (1.0)	8 (2.0)	.69	1 (1.1)	2 (2.2)	.56
Mesenteric	36 (7.2)	8 (8.0)	28 (7.1)	.74	7 (7.9)	7 (7.9)	1.0
Renal	33 (6.6)	9 (9.0)	24 (6.0)	.29	9 (10)	5 (5.6)	.27
Lower extremity	38 (7.6)	9 (9.0)	29 (7.3)	.57	8 (9.0)	9 (10)	.80
Delayed operation	50 (10)	13 (13)	37 (9.3)	.27	12 (13)	11 (12)	.82
Medications							
Anticoagulant within 48 h	56 (11)	12 (12)	44 (11)	.80	10 (11)	10 (11)	1.0
Aspirin within 5 d	109 (22)	18 (19)	91 (23)	.32	17 (20)	20 (23)	.61
Preoperative hemoglobin (g/dL)	12.5 (11.2, 13.8)	12.6 (11.6, 13.0)	11.6 (9.6, 13.5)	**<.0001**	12.0 (9.6, 13.6)	11.9 (10.5, 13.4)	.87
Preoperative hematocrit (%)	38 (34, 41)	38 (35, 41)	35 (30, 40)	**.0003**	35.5 (30.0, 40.2)	34.7 (31.8, 40.1)	.81
Preoperative platelets	177 (145, 219)	177 (148, 219)	173 (134, 224)	.61	182 (134, 224)	161 (138, 210)	.78
INR	1.1 (1.0, 1.1)	1.1 (1.0, 1.1)	1.1 (1.0, 1.2)	.22	1.1 (1.0, 1.2)	1.0 (1.0, 1.1)	**.04**

Values are presented as median (25%, 75%) for continuous data and n (%) for categorical data. Bolded *P* values indicate statistical significance. *ABT*, Autologous blood transfusion; *PSM*, propensity score match; *BMI*, body mass index; *CAD*, coronary artery disease; *COPD*, chronic obstructive pulmonary disease; *MI*, myocardial infarction; *CVA*, cerebrovascular accident; *PVD*, peripheral vascular disease; *CTD*, connective tissue disease; *AI*, aortic insufficiency; *INR*, international normalized ratio.

∗ *P* values indicate the difference between autologous blood transfusion and no autologous blood transfusion groups.

### Operative Data

Overall, both ABT and No-ABT groups underwent similar procedures, including root and concomitant procedures; however, the ABT group underwent more zone 1 arch replacements (11% vs 3.0%; *P* = .02). CPB, aortic crossclamp, and hypothermic circulatory arrest times were similar between groups as were cerebral perfusion strategy and lowest temperature. Significantly fewer patients in the ABT group needed blood transfusion (75% vs 92%; *P* < .001).

Among the propensity-matched cohort, the ABT and No-ABT groups underwent similar procedures with similar CPB, aortic crossclamp, and hypothermic circulatory arrest times as well as similar cerebral perfusion strategy and had similar lowest temperatures. Compared with the No-ABT group, the propensity-matched ABT group required less total intraoperative blood products (6 U [2, 11] vs 11 U [5, 17]; *P* < .0001), PRBCs (2 U [0, 4] vs 4 U [1, 8]; *P* = .001), FFP (2 U [0, 3] vs 4 U [1, 6]; *P* < .0001), platelets (2 U [0, 4] vs 2 U [1, 5]; *P* = .03), and cryoprecipitate (0 U [0, 2] vs 1 U [0, 2]; *P* = .005). The transfusion rates of FFP (57% vs 80%), platelets (66% vs 83%), and cryoprecipitate (33% vs 54%) were significantly lower in the ABT group in the No-ABT group. The use of activated factor VII was similar in the ABT (5.6%) and No-ABT (8.9%) groups.

### Postoperative Outcomes

Overall, in the whole cohort, fewer patients in the ABT group required blood transfusion (36% vs 52%; *P* = .004). The ABT group also had fewer postoperative complications, including reoperation for bleeding (3.3% vs 10%; *P* = .01), sepsis (1.8% vs 7.0%; *P* = .02), stroke (5.0% vs 13%; *P* = .004), acute renal insufficiency, acute renal failure requiring dialysis (6.3% vs 15%; *P* = .004), prolonged ventilation (48% vs 71%; *P* < .0001), reintubation (6.1% vs 12%; *P* = .04), tracheostomy (1.5% vs 5.0%; *P* = .0496), shorter postoperative lengths of stay (9 vs 13 days; *P* = .0002), and lower operative mortality (5.3% vs 15%; *P* = .0008).

**Figure 1:**
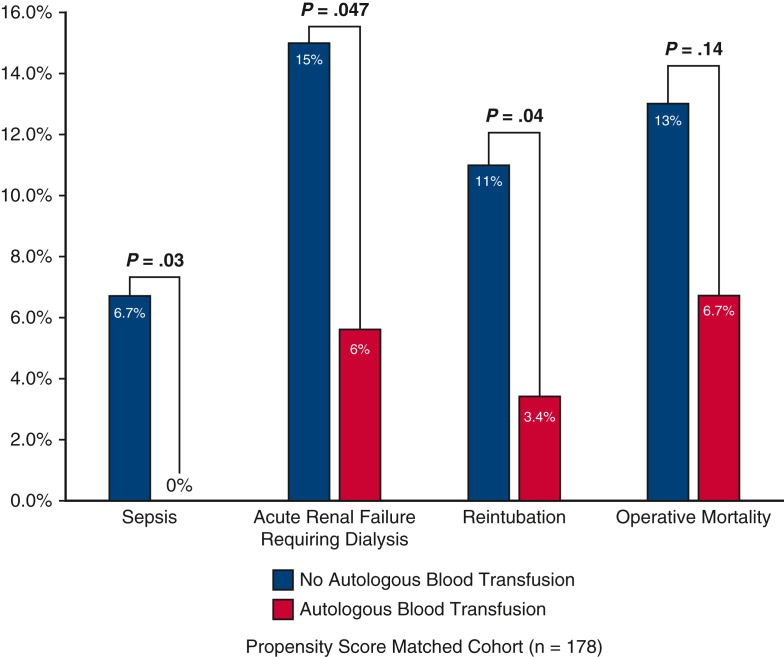
Patients with autologous blood transfusion had better postoperative outcomes, including sepsis, acute renal failure requiring dialysis, reintubation, and operative mortality.

Among the propensity-matched cohort, there was no significant difference in the rate of postoperative transfusion of blood products. However, the combined intraoperative and postoperative transfusion of any blood products was significantly lower in the ABT group compared with the No-ABT group (9 U [5, 13] vs 13 U [8, 22]; *P* < .001). The ABT group also had significantly less sepsis (0% vs 6.7%; *P* = .03), acute renal insufficiency, acute renal failure requiring dialysis (5.6% vs 15%; *P* = .047), shorter time intubated (37.5 vs 65.6 hours; *P* = .007), and shorter postoperative lengths of stay (9 vs 12 days; *P* = .02) (Table 2 and Figure 1).

**Table 2:** Postoperative data

Variable	Total (N = 497)	No-ABT (n = 100)	ABT (n = 397)	*P* value∗	PSM-No-ABT (n = 89)	PSM-ABT (n = 89)	*P* value∗
Any blood products	193 (39)	52 (52)	141 (36)	**.004**	43 (48)	35 (39)	.29
PRBCs	171 (34)	47 (47)	124 (31)	**.004**	38 (43)	27 (30)	.12
FFP	76 (15)	22 (22)	54 (14)	.05	17 (19)	12 (14)	.42
Platelets	87 (18)	26 (26)	61 (15)	**.02**	21 (24)	17 (19)	.58
Cryoprecipitate	22 (4)	7 (7)	15 (4)	.26	5 (7)	4 (5)	1
Combined intraoperative and postoperative transfusion							
Any blood products	415 (84)	96 (96)	319 (80)	**.0002**	84 (94)	76 (85)	.08
Blood transfused (U)	9.0 (4.0, 14)	14 (8.0, 23)	7 (4, 13)	**<.001**	13 (8.0, 22)	9.0 (5.0, 13)	**<.001**
Reoperation for bleeding	23 (4.6)	10 (10)	13 (3.3)	**.01**	5 (5.6)	2 (2.2)	.44
Tamponade	6 (1.2)	2 (2.0)	4 (1.0)	.35	1 (1.1)	2 (2.2)	.56
Deep sternal wound infection	2 (0.4)	0 (0)	2 (0.5)	.48	0 (0)	0 (0)	1.0
Sepsis	14 (2.8)	7 (7.0)	7 (1.8)	**.02**	6 (6.7)	0 (0)	**.03**
Postoperative MI	3 (0.6)	1 (1.0)	2 (0.5)	.49	1 (1.1)	1 (1.1)	1.0
Atrial fibrillation	169 (34)	40 (40)	129 (32)	.16	34 (38)	25 (28)	.15
Cerebrovascular accident	33 (6.6)	13 (13)	20 (5.0)	**.004**	10 (11)	5 (5.6)	.18
TIA	1 (0.2)	0 (0)	1 (0.3)	.62	0 (0)	0 (0)	1.0
New-onset paraplegia	5 (1.0)	1 (1.0)	4 (1.0)	1.0	1 (1.1)	1 (1.1)	1.0
Acute renal insufficiency	86 (17)	31 (31)	55 (14)	**<.0001**	27 (30)	12 (13)	**.007**
Acute renal failure†	71 (14)	25 (25)	46 (12)	**.0006**	21 (24)	10 (11)	**.03**
Requiring dialysis	40 (8.0)	15 (15)	25 (6.3)	**.004**	13 (15)	5 (5.6)	**.047**
Permanent	22 (4.4)	8 (8.0)	14 (3.5)	.06	7 (7.9)	4 (4.5)	.35
Gastrointestinal complications	45 (9.1)	13 (13)	32 (8.1)	.12	11 (12)	6 (6.7)	.20
Pneumonia	75 (15)	17 (17)	58 (15)	.55	14 (16)	10 (11)	.38
Prolonged ventilation‡	262 (53)	70 (71)	192 (48)	**<.0001**	59 (67)	48 (54)	.07
Hours intubated	37 (21, 85)	68 (31, 124)	32 (20, 68)	**<.0001**	65.6 (25.7, 120.6)	37.5 (22.4, 74.1)	**.007**
Reintubation	36 (7.2)	12 (12)	24 (6.1)	**.04**	10 (11)	3 (3.4)	**.04**
Tracheostomy	11 (2.2)	5 (5.0)	6 (1.5)	**.0496**	3 (3.4)	1 (1.1)	.62
Postoperative LOS (d)	10 (7.0, 16)	13 (8.0, 21)	9.0 (7.0, 14)	**.0002**	12 (8.0, 19)	9.0 (7.0, 14)	**.02**
Total LOS (d)	10 (7.0, 17)	15 (8.5, 25)	9.0 (7.0, 15)	**<.0001**	13 (8.0, 22)	9.0 (7.0, 17)	**.008**
Intraoperative mortality	2 (0.4)	2 (2.0)	0 (0)	**.04**	2 (2.2)	0 (0)	.50
In-hospital mortality	34 (6.8)	14 (14)	20 (5.0)	**.002**	11 (12)	6 (6.7)	.20
30-d mortality	29 (5.8)	10 (10)	19 (4.8)	**.047**	8 (9.0)	6 (6.7)	.58
Operative mortality§	36 (7.2)	15 (15)	21 (5.3)	**.0008**	12 (13)	6 (6.7)	.14

Values are presented as median (25%, 75%) for continuous data and n (%) for categorical data. Only patients who received blood transfusion were included to calculate the median use of blood products. Bolded *P* values indicate statistical significance. *ABT*, Autologous blood transfusion; *PSM*, propensity score match; *PRBC*, packed red blood cells; *FFP*, fresh frozen plasma; *MI*, myocardial infarction; *TIA*, transient ischemic attack; *LOS*, length of stay.

∗ *P* value indicates the difference between ABT and No-ABT groups.

† Acute renal failure defined using the Society of Thoracic Surgeons definition: an increase in serum creatinine level 3 times greater than baseline, or serum creatinine level ≥4 mg/dL, with an acute rise being at least 0.5 mg/dL and/or a new requirement for dialysis postoperatively.

‡ >24 hours.

§ Operative mortality includes 30-day mortality and/or in-hospital mortality.

Peripheral vascular disease (odds ratio [OR], 2.57; 95% CI, 1.34-4.96; *P* = .005) was a significant risk factor for any blood product transfusion (intraoperative + postoperative), whereas ABT (OR, 0.28; 95% CI, 0.11-0.75; *P* = .01) and body mass index (OR, 0.95; 95% CI, 0.91-0.98; *P* = .004) were protective against any blood product transfusion. Preoperative renal failure (OR, 4.20; 95% CI, 1.67-10.6; *P* = .002), acute stroke (OR, 4.55; 95% CI, 1.71-12.1; *P* = .002), and cardiogenic shock (OR, 6.13; 95% CI, 2.55-14.8; *P* < .0001) were significant risk factors for operative mortality (Table 3).

**Table 3:** Risk factors for any transfusion of blood products intra- or postoperatively and operative mortality in the whole cohort by multivariable logistic model

Risk factor	Odds ratio (95% CI)	*P* value
Blood transfusion		
ABT	0.28 (0.11-0.75)	.01
Sex, male	0.69 (0.36-1.31)	.25
Age	1.02 (1.00-1.04)	.06
BMI	**0.95 (0.91-0.98)**	**.004**
PVD	**2.57 (1.34-4.96)**	**.005**
Previous cardiac surgery	1.61 (0.45-5.77)	.46
Preoperative renal failure	1.67 (0.67-4.19)	.27
Cardiogenic shock	2.59 (0.86-7.78)	.09
Preoperative hemoglobin	0.87 (0.74-1.02)	.08
Any CTD	0.96 (0.18-5.27)	.96
Operative mortality		
ABT	0.52 (0.22-1.21)	.13
Male sex	0.81 (0.68-4.83)	.24
Age	1.01 (0.98-1.04)	.50
COPD	1.35 (0.49-3.84)	.57
Prior cardiac surgery	2.23 (0.64-7.81)	.21
Preoperative renal failure	**4.20 (1.67-10.6)**	**.002**
Acute stroke	**4.55 (1.71-12.1)**	**.002**
Acute paralysis	3.95 (0.96-18.1)	.08
Cardiogenic shock	**6.13 (2.55-14.8)**	**<.0001**
Any CTD	2.5 (0.36-17.4)	.35

Bolded *P* values indicate statistical significance. *CI*, Confidence interval; *ABT*, autologous blood transfusion; *BMI*, body mass index; *PVD*, peripheral vascular disease; *CTD*, connective tissue disease; *COPD*, chronic obstructive pulmonary disease.

### Midterm Outcomes

**Figure 2:**
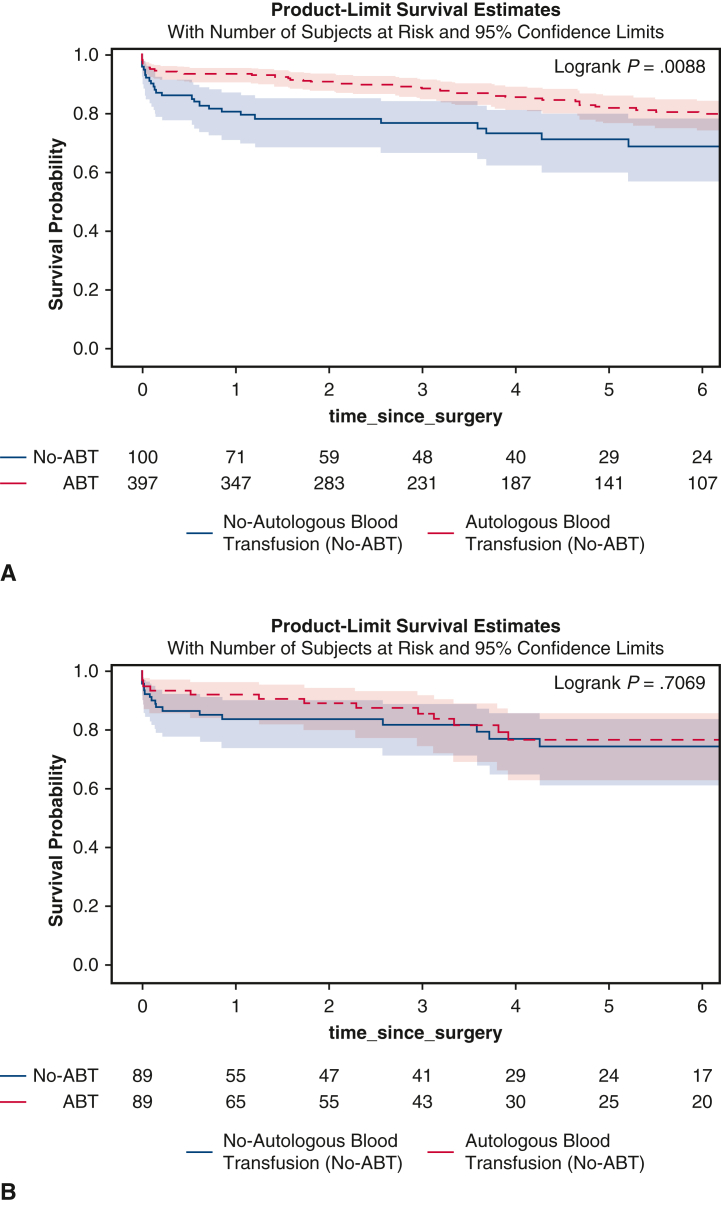
Kaplan-Meier survival analysis of patients undergoing open acute type A aortic dissection repair with or without autologous blood transfusion (*ABT*) before (A) and after (B) propensity score match. The ABT group had significantly better midterm survival (5 year: 82% [95% CI, 77%-86%] vs 69% [95% CI, 57%-78%]; *P* = .009) in the whole cohort (A), but not in the propensity score-matched cohort (5 year: 76% [95% CI, 63%-85%] vs 74% [95% CI, 61%-84%]; *P* = .71) (B).

The mean follow-up time was 4.1 ± 3.0 years. The 5-year survival from date of surgery was better in the ABT compared with the No-ABT groups (82% [95% CI, 77%-86%] vs 69% [95% CI, 57%-79%]; *P* = .009) (Figure 2, *A*). However, there was no significant difference in midterm survival between the propensity score matching groups (76% [95% CI, 63%-85%] vs 74% [95% CI, 61%, 84%]; *P* = .71) (Figure 2, *B*). Preoperative renal failure (HR, 2.52; 95% CI, 1.43, 4.43; *P* = .001) and cardiogenic shock (HR, 2.87; 95% CI, 1.67-4.93; *P* = .0001) were significant risk factors for midterm mortality among the whole cohort; ABT did not significantly affect midterm survival (HR, 0.81; 95% CI, 0.49-1.3; *P* = .41) (Table 4).

**Table 4:** Risk factors for long-term mortality in the whole cohort by multivariable cox model

Risk factor	Hazard ratio (95% CI)	*P* value
ABT	0.81 (0.49-1.3)	.41
Age	1.038 (1.02-1.06)	<.0001
Male sex	1.68 (1.02-2.80)	**.047**
COPD	1.70 (0.97-2.98)	.06
Previous cardiac surgery	1.56 (0.76-3.18)	.22
Preoperative renal failure	**2.52 (1.43-4.43)**	**.0013**
Acute paralysis	1.31 (0.45-3.83)	.63
Acute stroke	1.70 (0.88-3.29)	.11
Cardiogenic shock	**2.87 (1.67-4.93)**	**.0001**
Any CTD	1.72 (0.48-6.16)	.41
Diabetes	1.16 (0.60-2.24)	.65

Bolded *P* values indicate statistical significance. *CI*, Confidence interval; *ABT*, autologous blood transfusion; *COPD*, chronic obstructive pulmonary disease; *CTD*, connective tissue disease.

## Discussion

In this study, we found autologous blood transfusion significantly decreased intraoperative blood transfusion as well as combined intraoperative and postoperative blood transfusion, and improved the perioperative outcomes in ATAAD repair, including postoperative sepsis, acute renal failure, time of intubation and reintubation, and hospital length of stay. The operative mortality decreased by half (6.3% vs 13%; *P* = .14) with ABT, but the midterm survival was similar in patients with or without ABT after propensity score match (Video 1).

The STS/Society of Cardiovascular Anesthesiologists clinical practice guidelines on perioperative blood transfusion and blood conservation in cardiac surgery identify 6 risk factors associated with increased blood transfusion: advanced age, low preoperative red blood cell volume (preoperative anemia or small body size), preoperative antiplatelet or antithrombotic drugs, reoperative or complex procedures, emergency operations, and noncardiac patient comorbidities.^14^ ATAAD repair is an emergency and complex procedure. Not surprising, 93.7% of patients received some form of blood product in a recent STS database study.^5^ There is a demand to improve transfusion rate in ATAAD repair.

At our institution, we started to use ABT in elective cardiac cases, such as coronary artery bypass, aortic valve replacement, and aortic cases 2 decades ago. This approach dramatically reduced our transfusion rate. We gradually adapted this approach to emergency operations of ATAAD. There was always a concern for safety of harvesting the autologous blood. Some surgeons and anesthesiologists were aggressive on taking autologous blood, whereas some were more conservative. After observing more and more patients tolerating the ABT well with excellent outcomes, it has become a routine approach to harvest ABT for all patients with ATAAD at present except in cases that require crashing on CPB or with severe anemia (hemoglobin <9 g/dL). In this study, 80% of patients with ATAAD had autologous blood harvested and transfused, which was more than reported in the literature^8^ (22% patients with ATAAD received autologous platelets enriched plasma). We did not see any complications from autologous blood harvesting in patients with ATAAD, even in those with cardiac tamponade. We normally relieved the cardiac tamponade then harvested the autologous blood when patients were more stable. The only patients we do not harvest autologous blood from are those who need immediate CPB for survival or with a hemoglobin level <9 g/dL. Sometimes, patients became hypotensive due to volume loss during the autologous blood harvest, which was managed with intravascular volume repletion with crystalloids. The approach of ABT in patients with ATAAD during open aortic repair has been very safe in our practice.

The benefits of ABT were very clear. The intraoperative transfusion of total blood products and components of the blood were decreased by half or more in the ABT group (Table 5), which was consistent with previous reports by other groups using autologous platelet enriched plasma^8^ or blood.^6^^,^^15^ Subsequently, the ABT group had significantly less postoperative PRBC transfusion and well-known transfusion-associated complications, such as sepsis due to immune suppression from blood transfusion, acute renal failure due to kidney injury from blood transfusion, intubation time and reintubation due to transfusion-associated acute lung injury, and length of stay (Table 2 and Figure 1) The operative mortality was doubled in the non-ABT group after propensity score match, although not statistically significant most likely due to small sample size of the matched cohort, a downside of a propensity score matched analysis. Other groups have also reported the intraoperative PRBC transfusion was associated with operative mortality.^3^^,^^16^^,^^17^ The cause of operative mortality in ATAAD repair is very complex due to the complexity of the disease. A direct cause-effect link between ABT and better early survival was difficult to establish at this stage. To our surprise, the ABT did not affect midterm survival based on Kaplan-Meier analysis (Figure 2, *B*) after propensity score match and multivariable Cox model (Table 4). Another study in cardiac surgery also showed transfusion of PRBCs was not associated with midterm mortality.^18^ The benefit of ABT appeared to be better short-term outcomes.

**Table 5:** Intraoperative data

Variable	Total (N = 497)	No-ABT (n = 100)	ABT (n = 397)	*P* value∗	PSM-No-ABT (n = 89)	PSM–ABT (n = 89)	*P* value∗
Aortic root procedure				.82			.82
None	36 (7.2)	9 (9.0)	27 (6.8)	.45	8 (9.0)	5 (5.6)	.39
AVR only	12 (2.4)	2 (2.0)	10 (2.5)	.76	2 (2.2)	1 (1.1)	.56
Root replacement	153 (31)	33 (33)	120 (30)	.59	29 (33)	27 (30)	.75
Root repair	296 (60)	56 (56)	240 (60)	.42	50 (56)	56 (63)	.36
Arch replacement				.14			.23
None	22 (4.4)	4 (4.0)	18 (4.5)	.82	3 (3.4)	6 (6.7)	.30
Hemiarch	309 (62)	64 (64)	245 (62)	.67	56 (63)	58 (65)	.75
Zone 1 Arch	46 (9.3)	3 (3.0)	43 (11)	**.02**	3 (3.4)	8 (9.0)	.12
Zone 2 Arch	91 (18)	23 (23)	68 (17)	.17	22 (25)	13 (15)	.09
Zone 3 Arch	29 (5.8)	6 (6.0)	23 (5.8)	.94	5 (5.6)	4 (4.5)	.73
Frozen elephant trunk	99 (20)	16 (16)	83 (21)	.27	14 (16)	18 (20)	.43
Concomitant procedures							
CABG	25 (5.0)	6 (6.0)	19 (4.8)	.62	5 (5.6)	6 (6.7)	.76
Mitral valve	8 (1.6)	1 (1.0)	7 (1.8)	.59	0 (0)	1 (1.1)	.32
Tricuspid valve	6 (1.2)	1 (1.0)	5 (1.3)	.83	0 (0)	1 (1.1)	.32
CPB time (min)	218 (177, 269)	227 (183, 289)	217 (176, 265)	.15	227 (191, 289)	217 (182, 253)	.19
Crossclamp time (min)	142 (110, 198)	148 (116, 195)	142 (109, 199)	.53	150 (120, 192)	139 (110, 196)	.50
HCA	477 (96)	96 (96)	381 (96)	.99	86 (97)	84 (94)	.47
HCA time (min)	30 (22, 39)	29 (23, 42)	31 (22, 39)	.89	30 (23, 43)	31 (24, 38)	.66
Cerebral perfusion				.97			.79
None	20 (4.0)	4 (4.0)	16 (4.0)	.99	3 (3.4)	5 (5.6)	.47
Antegrade	308 (62)	64 (64)	244 (61)	.64	59 (66)	56 (63)	.64
Retrograde	117 (24)	22 (22)	95 (24)	.68	18 (20)	21 (24)	.59
Both antegrade and retrograde	52 (10)	10 (10)	42 (11)	.87	9 (10)	7 (7.9)	.60
Lowest temperature (°C)	20 (18, 24)	19 (18, 24)	20 (18, 24)	.43	18.5 (18, 24)	19.8 (18, 24)	.37
Lowest HCT (%) on CPB	21 (18, 23)	20 (18, 24)	21 (19, 23)	.06	20 (18, 24)	21 (18, 23)	.92
Any blood products	390 (79)	92 (92)	298 (75)	**<.001**	81 (91)	73 (82)	.12
Blood transfused (U)	6.0 (1.0, 11)	12 (6.5, 18)	4.0 (1.0, 9.0)	**<.0001**	11 (5.0, 17)	6.0 (2.0, 11)	**<.0001**
PRBCs	305 (61)	84 (84)	221 (56)	**<.001**	73 (82)	63 (71)	.11
PRBCs (U)	1.0 (0.0, 4.0)	4.0 (1.0, 8.0)	1.0 (0.0, 3.0)	**<.0001**	4.0 (1.0, 8.0)	2.0 (0.0, 4.0)	**.001**
FFP	287 (58)	81 (81)	206 (52)	**<.001**	71 (80)	51 (57)	**.002**
FFP (U)	1.0 (0.0, 4.0)	4.0 (1.0, 8.0)	1.0 (0.0, 3.0)	**<.0001**	4.0 (1.0, 6.0)	2.0 (0.0, 3.0)	**<.0001**
Platelets	330 (66)	85 (85)	245 (62)	**<.001**	74 (83)	59 (66)	**.02**
Platelets (U)	2.0 (0.0, 3.0)	3.0 (1.0, 5.0)	2.0 (0.0, 3.0)	**<.0001**	2.0 (1.0, 5.0)	2.0 (0.0, 4.0)	**.03**
Cryoprecipitate	171 (34)	55 (55)	116 (29)	**<.001**	48 (54)	29 (33)	**.006**
Cryoprecipitate (U)	0.0 (0.0, 2.0)	1.0 (0.0, 2.0)	0.0 (0.0, 1.0)	**<.0001**	1.0 (0.0, 2.0)	0.0 (0.0, 2.0)	**.005**
Autologous blood volume (mL)	900 (900, 1350)	–	900 (900, 1350)	–	–	900 (450, 1350)	–
Activated factor VII	34 (6.8)	9 (9.0)	25 (6.3)	.34	8 (8.9)	5 (5.6)	.57

Values are presented as median (25%, 75%) for continuous data and n (%) for categorical data. Only patients who received blood transfusion. Bolded *P* values indicate statistical significance. *ABT*, Autologous blood transfusion; *PSM*, propensity score match; *AVR*, aortic valve replacement; *CABG*, coronary artery bypass graft; *CPB*, cardiopulmonary bypass; *HCA*, hypothermic circulatory arrest; *HCT*, hematocrit; *PRBCs*, packed red blood cells. *FFP*, fresh frozen plasma.

∗ *P* values indicates the difference between autologous blood transfusion and no autologous blood transfusion groups.

Given that patients in the No-ABT group likely did not undergo harvesting due to aortic rupture, cardiac arrest with immediate CPB, low preoperative hemoglobin level, or were otherwise unstable, one could argue that patients in the No-ABT group were sicker to start with. We agree that surgeons and anesthesiologists would be hesitant to harvest autologous blood in unstable patients with cardiac tamponade and cardiogenic shock. Before propensity score match, the non-ABT group did have significantly more cardiogenic shock (24% vs 7.3%), which reflects the concern of the team to harvest the autologous blood. However, after propensity score match, the ABT group had a similar proportion of patients with cardiogenic shock compared to No-ABT group (21% vs 17%; *P* = .45) and other demographic variables (Table 1) Although INR was statistically longer in the No-ABT group, we did not think an INR of 1.1 was very clinically different from an INR of 1.0. The intraoperative data were very similar between the 2 groups before and after propensity score match, indicating the intraoperative factors had similar effects on outcomes in both groups. Taken together, we believed after propensity score match, the 2 groups were very comparable, and the results from the propensity score match analysis reflected the effect of ABT on the outcomes of ATAAD repair.

This study is limited by its retrospective nature, the relatively small sample size, and that it is not a randomized trial. Because we have seen the tremendous benefit of ABT, we (ie, surgical team and anesthesia team) routinely harvest autologous blood for all patients with ATAAD unless it is absolutely contraindicated. This makes it difficult to run a randomized trial. The sample size of patients not undergoing ABT became smaller after propensity score match, which reduced the statistical power and increased type II error. The 2 groups were not completely balanced even after propensity score match. The transfusion criteria for PRBCs was a hematocrit of 18% in our study. The transfusion of platelets, FFP, and cryoprecipitate depended on the surgeon's judgment of coagulopathy, which was subjective. However, the primary outcomes for this study were transfusion of PRBCs and perioperative complications. Because of the limitations, additional external validation in other cohorts before general employment of ABT in ATAAD repair is needed.

## Conclusions

**Figure 3:**
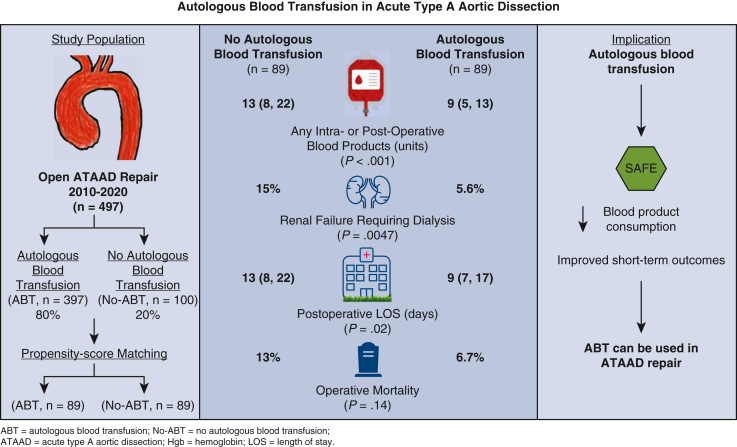
Autologous blood harvesting and transfusion was safe and improved short-term outcomes in patients undergoing open acute type A aortic dissection (*ATAAD*) repair. All patients undergoing open ATAAD repair could have autologous blood transfusion (*ABT*) unless they are unstable needing immediate cardiopulmonary bypass or severely anemic (hemoglobin <9 g/dL).

Autologous blood harvesting and transfusion was safe and improved short-term outcomes in patients with ATAAD undergoing open repair. This practice could be applied for all patients with ATAAD in general, but external validation in a multicenter setting is needed (Figure 3).

### Conflict of Interest Statement

The authors reported no conflicts of interest.

The *Journal* policy requires editors and reviewers to disclose conflicts of interest and to decline handling or reviewing manuscripts for which they have a conflict of interest. The editors and reviewers of this article have no conflicts of interest.

## Supplementary Data

Video 1 Discussion of the influence of autologous blood transfusion on the outcomes of acute type A aortic dissection repair. Video available at: https://www.jtcvs.org/article/S2666-2736(22)00300-X/fulltext.
