# Supplementary figures and images for: Autologous blood transfusion in acute type A aortic dissection decreased blood product consumption and improved postoperative outcomes
Source: JTCVS Open. 2022 Jul 20;12:20–9. doi: 10.1016/j.xjon.2022.07.005 (PMC9801237; doi:10.1016/j.xjon.2022.07.005)

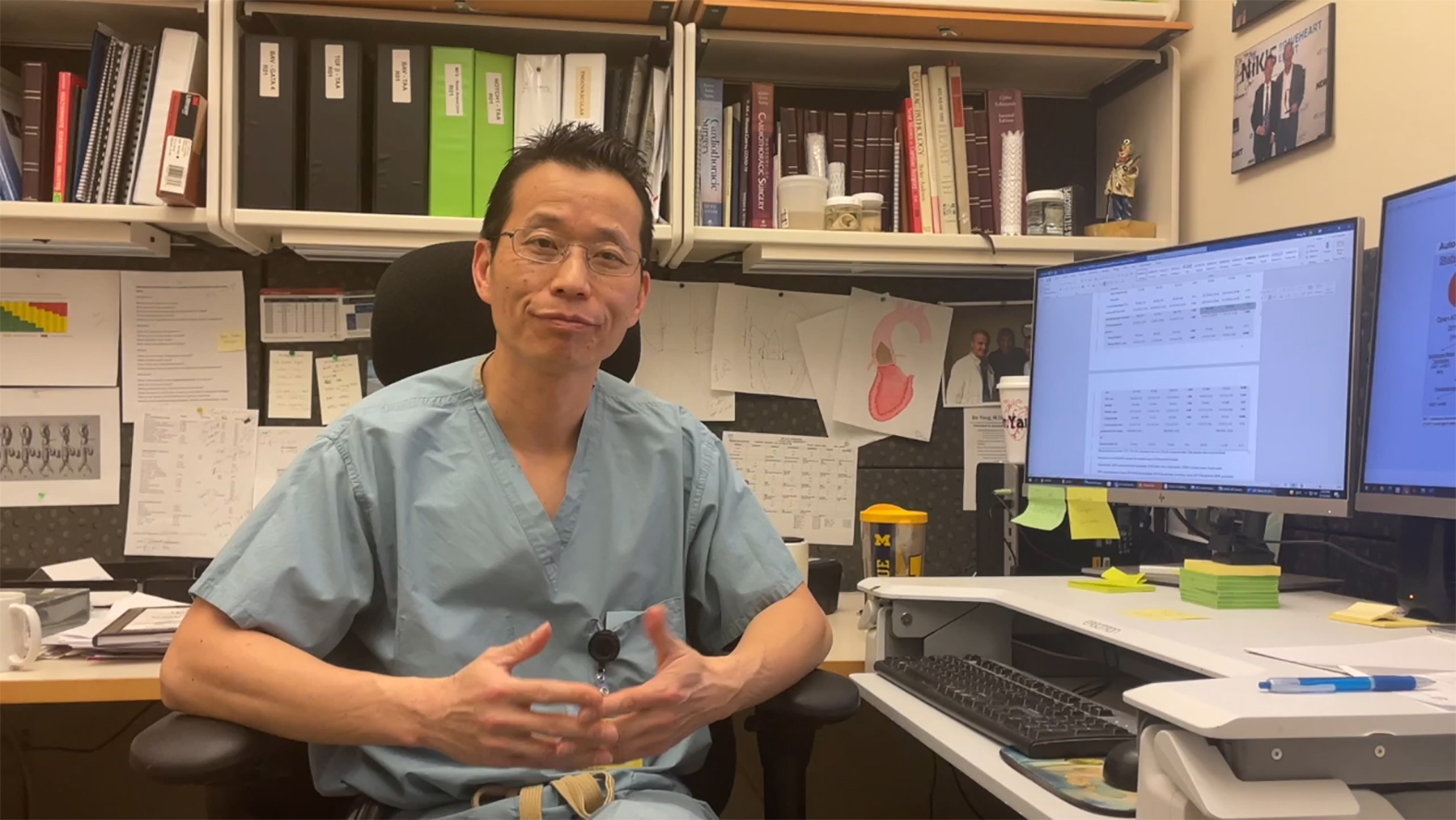

Supplement: Video 1 — Discussion of the influence of autologous blood transfusion on the outcomes of acute type A aortic dissection repair. Video available at: https://www.jtcvs.org/article/S2666-2736(22)00300-X/fulltext. [file fx3.jpg]
